# Influence of Gut Microbiota on Progression to Tuberculosis Generated by High Fat Diet-Induced Obesity in C3HeB/FeJ Mice

**DOI:** 10.3389/fimmu.2019.02464

**Published:** 2019-10-18

**Authors:** Lilibeth Arias, Galo Adrián Goig, Paula Cardona, Manuela Torres-Puente, Jorge Díaz, Yaiza Rosales, Eric Garcia, Gustavo Tapia, Iñaki Comas, Cristina Vilaplana, Pere-Joan Cardona

**Affiliations:** ^1^Experimental Tuberculosis Unit (UTE), Fundació Institut Germans Trias i Pujol (IGTP), Badalona, Spain; ^2^Centro de Investigación Biomédica en Red de Enfermedades Respiratorias (CIBERES), Madrid, Spain; ^3^Department of Genetics and Microbiology, Universitat Autònoma de Barcelona (UAB), Badalona, Spain; ^4^Tuberculosis Genomics Unit (TGU), Instituto de Biomedicina de Valencia (IBV-CSIC), Valencia, Spain; ^5^Pathology Department, Hospital Universitari Germans Trias i Pujol (HUGTIP), Universitat Autònoma de Barcelona (UAB), Badalona, Spain; ^6^Centro de Investigación Biomédica en Red de Epidemiologia y Salud Pública (CIBERESP), Madrid, Spain

**Keywords:** tuberculosis, high fat diet, obesity, comorbidity, BCG, mice, gut microbiota, C3HeB/FeJ

## Abstract

The administration of a high fat content diet is an accelerating factor for metabolic syndrome, impaired glucose tolerance, and early type 2 diabetes. The present study aims to assess the impact of a high fat diet on tuberculosis progression and microbiota composition in an experimental animal model using a C3HeB/FeJ mouse strain submitted to single or multiple consecutive aerosol infections. These models allowed us to study the protection induced by Bacillus Calmette-Guérin vaccination as well as by the natural immunity induced by chemotherapy after a low dose *Mycobacterium tuberculosis* infection. Our results show that a high fat diet is able to trigger a pro-inflammatory response, which results in a faster progression toward active tuberculosis and an impaired protective effect of BCG vaccination, which is not the case for natural immunity. This may be related to dysbiosis and a reduction in the Firmicutes/Bacteroidetes ratio in the gut microbiota caused by a decrease in the abundance of the Porphyromonadaceae family and, in particular, the *Barnesiella* genus. It should also be noted that a high fat diet is also related to an increase in the genera *Alistipes, Parasuterella, Mucispirillum*, and *Akkermansia*, which have previously been related to dysbiotic processes. As diabetes mellitus type 2 is a risk factor for developing tuberculosis, these findings may prove useful in the search for new prophylactic strategies for this population subset.

## Introduction

Tuberculosis (TB) is still a major challenge facing humanity in the twenty-first century. Since the declaration by the World Health Organization of a global emergency 25 years ago, the absolute number of cases seems to have stabilized. However, the reduction in incidence of around 1.5% is far from the value of 25% needed to achieve the 2025 objective (1).

Several factors, including severe immunosuppression (caused by HIV, immunosuppressive drugs or anti-TNF antibodies), alcoholism and tobacco smoking, have been reported to increase the probability of developing TB (2). One of the most important factors is malnutrition (3, 4). A reduction in body mass index (BMI) to below 18 has been shown to be a strong risk factor for TB (5). In several infectious diseases, such as community-acquired pneumonia, sepsis, Chagas disease, or TB, there are consistent epidemiological data showing the advantages of obesity, with negative outcomes being inversely related to BMI. This benefit can also be seen for a variety of surgical and non-surgical conditions (6, 7). This means that subjects with better nutrition, more body fat and highly activated immune functions are more likely to survive. Metabolic syndrome intensifies with an increase in adiposity, thereby raising the level of immune protection, which at the same time is counterbalanced by the deleterious effects of dysglycemia. This process is known as the “obesity paradox” (6).

In contrast, it is well-known that diabetes mellitus type 2 (DM2) is also a risk factor for TB (8), although this is also linked to obesity and, therefore, an increased BMI. This new link is specially worrying considering the overwhelming increase in DM2 incidence in high TB burden countries, such as India and South Africa (9, 10). Obesity, insulin resistance and DM2 are closely associated with a chronic state of low-grade inflammation (11). In particular, in obesity there is an over-expression of TNF-α, which attracts M1 macrophages, thereby increasing the levels of inflammatory mediators such as C-reactive protein, IL-1, IL-6, IL-8, IL-12, and IFN-γ. In this sense, DM2 also results in an increase in neutrophils and monocyte-secreted cytokines, including TNF-α, IL-6, and IL-8. In addition, there is an impairment in infection-induced cytokine production and a reduction in phagocytic and antibacterial activity. DM2 decreases the level of regulatory T cells (Tregs) while increasing Th17, thus promoting an exaggerated inflammatory response (12).

A high fat diet (HFD) has been linked to an increased diversity in the intestinal microbiota, thus providing an anti-inflammatory milieu (13), and a decrease in the Firmicutes to Bacteroidetes (F/B) ratio, which has been related to glucose intolerance (14). In an experimental model of TB using BALB/c mice, infection induced an initial decrease in microbiota diversity, which tended to recover with chronification. This work highlights the significant decrease in Clostridiales (phylum Firmicutes), as promoters of Tregs, and Bacteroidales (phylum Bacteroidetes), as inducers of IL-10-producing T cells and modulators of the Th1/Th2 balance (15). Recent studies of an experimental model in C57BL/6J-CD45a mice infected with *Mycobacterium tuberculosis* (*Mtb*) demonstrated the rapid dysbiosis caused by conventional TB chemotherapy, with this condition persisting for a long time after cessation (16). The impact of standard treatment (HRZE) in TB patients has also been assessed, demonstrating a long-lasting microbiota dysbiosis with a variation in several species of the Firmicutes and Bacteroidetes phyla (17). Interestingly, both studies showed an increased abundance of the Erysipelotrichaceae family, which, in contrast to other Firmicutes, has been linked to a pro-inflammatory milieu (18). Curiously, a recent study in a human cohort has shown an increase in the gut microbiota diversity in TB patients. This is an interesting finding given that the BMI of TB patients was significantly lower than for healthy controls (19).

The first objective of this study was to evaluate the role of HFD-induced obesity in the development of TB in an active TB murine model using the C3HeB/FeJ mouse strain (20) by assessing the bacillary load (BL), pathology and cytokine profiles in lungs, survival rates, and gut microbiota content. We also evaluated the effect of multiple consecutive infections vs. a single aerosol infection. This is a better way of mimicking the scenario of household contacts, where subjects are at high risk of developing TB due to their daily contact with a TB patient and those persons that live in regions with a high infection risk (21). In this complex scenario, we tested the influence of Bacillus Calmette-Guérin (BCG) vaccination and natural infection on disease progression.

As there is a lack of animal studies regarding the mechanisms behind the DM2-TB comorbidity, which are key to better understanding the pathology and improving the management of the disease, we believe this research represents a significant contribution to the field by providing an animal model for testing TB vaccines in a context of obesity and early DM2, an issue closely related to current society.

## Materials and Methods

### Experimental Design

Two different experiments were planned in order to evaluate: (a) the effect of HFD-induced obesity on the mouse model of active TB using the C3HeB/FeJ strain (comorbidity model), as well as BCG vaccination and two different types of challenge [single infection or multiple consecutive infections (SI or MCI)]; and (b) the effect of BCG vaccination compared to natural immunity (NI) in the same comorbidity model.

Table 1 shows the experimental design of both experiments and Table 2 the experimental groups and experimental conditions used.

**Table 1:** Experimental design of both experiments performed in the study.

**Experimental design**
**Experiment 1**	**Time of infection**	**–w12**	**w0**	**w3**	**w4**	**w16**	**w16 → **
	**Time of diet**	**w0**	**w12**	**w15**	**w16**	**w28**	**w28 →**
		BCG	Infection	Euthanasia (*n* = 5)	Euthanasia (*n* = 5)	Euthanasia (*n* = 10)	Survival (*n* = 10)
**Experiment 2**	**Time of infection**	**–w12**	**–w10 to w0**	**w0**	**w0 →**		
	**Time of diet**	**w0**	**w2 to w10**	**w12**	**w12 →**		
		Single infection	Antibiotic treatment	Infection	Survival (*n* = 10)		

*Bold values correspond to weeks post and pre-infection*.

*–w12: 12 weeks before challenge; w3, w4, w16: 3, 4, and 16 weeks post-challenge*.

**Table 2:** Experimental groups and conditions tested per experiment.

**Experiment 1**				**Experiment 2**			
**Group**	**Experimental conditions**			**Group**	**Experimental conditions**		
	**Obesity**	**BCG**	**Type of infection**		**Obesity**	**Protection**	**Type of infection**
ND-SI	No	No	SI	**ND-NI**	NO	NI	MCI
ND-MCI	No	No	MCI	**HFD-NI**	YES	NI	MCI
ND-BCG-SI	No	Yes	SI				
ND-BCG-MCI	No	Yes	MCI				
HFD-SI	Yes	No	SI				
HFD-MCI	Yes	No	MCI				
HFD-BCG-SI	Yes	Yes	SI				
HFD-BCG-MCI	Yes	Yes	MCI				

In the first experiment (*n* = 240), four groups were subcutaneously vaccinated with 10^6^ colony-forming units (CFU) of BCG Live U.S.P vaccine (SII-ONCO-BCG®); the four remaining groups were sham vaccinated. After 12 weeks, mice were challenged with a low dose aerosol to deliver around 50 CFU of *M. tuberculosis* H37Rv Pasteur strain to the lungs using an airborne infection apparatus (Glas-col Inc., Terre Haute, IN, USA). In the case of MCI, this challenge was done by infecting animals eight times over a period of 5 days. Animals were euthanized at week 3 (*n* = 5 per group), week 4 [*n* = 5 per group, except for group normal diet (ND) and MCI with *n* = 4] and week 16 (*n* = 10 per group) post-infection (p.i.). BL and pathology in lungs were analyzed. Lung samples at week 4 were also used to evaluate immune responses. Fecal samples (*n* = 5 per group) were obtained at weeks 4 and 16 in order to analyze microbiota composition. The 10 remaining animals per group were allocated to assess impact on survival.

In the second experiment, the protective effect of BCG vaccination in MCI mice obtained from experiment 1 was compared to NI in MCI animals (*n* = 30). NI was used to evaluate the memory-immune response protection after *M. tuberculosis* challenge. To evaluate NI, animals were challenged with a SI of low dose aerosol and animals were left 2 weeks to develop immunity. Then, animals were treated with isoniazid and rifapentine (25 and 10 mg/kg, respectively) for 10 weeks from week 2 p.i., to sterilize lungs. In order to test whether undetectable BL was achieved, three animals of each group were euthanized and lung samples were plated. No CFU counts were detected after 21 days of incubation period (limit of detection = 10 CFU) (data not shown) as described in previous works (22–24). Two weeks after stopping antibiotic treatment, animals were challenged with MCI. Fecal samples (*n* = 5 per group) were obtained at week 4 in order to analyze microbiota composition. Differences in protection were assessed by survival (*n* = 10 per group).

### Animals

Female C3HeB/FeJ specific-pathogen-free mice (6–8 weeks old) were obtained from Jackson Laboratories (Bar Harbor, Maine, USA), and all procedures were conducted in a BSL-3 facility. Animals were maintained on a 12 h light-dark cycle in a temperature- and humidity-controlled room. Animals from non-obese experimental groups were fed with ND containing 13% calories from fat (2014S Teklad Global 14% Protein Rodent Maintenance Diet, Envigo). Animals from those experimental groups in which obesity was assessed were fed with an HFD containing 60.3% calories from fat [TD.06414 Adjusted Calories Diet (60/Fat), Envigo]. Animal weight was recorded weekly.

### Ethics

All procedures were performed according to protocol DMAH6119, which was reviewed by the Animal Experimentation Ethics Committee of the Hospital Universitari Germans Trias i Pujol (registered as B9900005) and approved by the Departament d'Agricultura, Ramaderia, Pesca, Alimentació i Medi Natural of the Catalan Regional Government, according to current national and European Union legislation regarding the protection of experimental animals. Mice were supervised daily following a strict monitoring protocol in order to ensure animal welfare, and euthanized, if required, with isoflurane (inhalation excess).

### Oral Glucose Tolerance Test

Oral glucose tolerance test was performed 11 weeks after diet feeding. After overnight fasting period, mice were administered with 2 g/kg by oral gavage. Blood samples were taken at 0, 15, 30, 60, and 120 min and glucose levels were measured with a glucometer (Accutrend Plus, Roche Diagnostics, Switzerland).

### Bacillary Load

Left lung samples from each animal were collected, homogenized and several dilutions plated on nutrient Middlebrook 7H11 agar (BD Diagnostics, Spark, USA). Visible CFU were counted after incubation for 28 days at 37°C.

### Lung Pathology

Right lower lung lobe samples were fixed in 10% buffered formalin, embedded in paraffin and 5 μm sections stained with hematoxylin-eosin for microscopic observation and analysis of the damaged area using the NISElements D version 3.0 × software package (Nikon Instruments Inc., Tokyo, Japan). Four recuts of a block containing all group samples were used to determine the damaged area as a percentage of total lung area. Moreover, the proportion of exudative lesions in the total damaged area was measured to obtain the relative exudative area.

### Survival

The effect on survival was evaluated in 10 animals from each group. Animals were euthanized according to the humanized end-point protocol approved by the ethics committee.

### Cytokine Profile Analysis

A cytokine profile study was performed in lung homogenates from right upper and middle lobes. The following cytokines were measured by Luminex xMAP® technology: IFN-γ, TNF-α, transforming growth factor beta (TGF-β), IL-2, IL-6, IL-10, IL12, IL-17, CXCL1, and CXCL5. Results are expressed as pg per ml of supernatant. The assay was performed with the MILLIPLEX® MAP kit (EMD Millipore Corporation, Billerica, MA, USA) following the manufacturer's instructions and analyzed with xPONENT Software (Luminex Corporation, Austin, TX, USA).

### Microbiota Analysis

#### Sample Processing and DNA Extraction

Cecum from the animals was extracted and stored at −80°C until DNA extraction. Samples were thawed on ice and stools separated from the intestinal epithelia. DNA was extracted using a QIAamp Fast DNA Stool Mini Kit (QIAGEN GmbH, Germany) according to the manufacturer's instructions. Three 4 mm glass beads were added in the first step before vortexing to enhance cell lysis. The total volume of eluate was stored at −20°C until concentration measurement. DNA concentrations of the extracts were measured fluorometrically using the Qubit dsDNA HS assay kit (Thermo Fisher Scientifics, Waltham, MA, USA). DNA was stored at −20°C until 16s rRNA gene library preparation.

#### 16S rRNA Gene Sequencing

Microbial DNA was analyzed using the Illumina *16S Metagenomic Sequencing Library Preparation* guide, with some modifications. The protocol targets the V3–V4 regions of the 16S rRNA gene, which were amplified using the KAPA HiFi HotStart PCR Kit (KAPA Byosystems, Wilmington, MA, USA). The composition of a typical PCR was as follows: 1X KAPA HiFi Fidelity Buffer, 0.5 mM MgCl_2_, 0.3 mM KAPA dNTP Mix, 0.3 μM primers, 0.5 U KAPA HiFi HotStart DNA Polymerase, and 15 ng DNA template. The PCR program with Mastercycler pro (Eppendorf, Hamburg, Germany) consisted of the following steps: initial denaturation at 95°C for 3 min, 18 cycles at 95°C for 30 s, 55°C for 30 s, and 72°C for 30 s, and a final extension at 72°C for 4 min. The expected product size was ~550 bp. The PCR products were purified using Agencourt AMPure XP magnetic beads (Beckman Coulter, Brea, CA) using a Magnetic Stand-96 (Thermo Fisher Scientific). The PCR products were quality controlled with a 1.4% Agarose Gel. The Index PCR was performed using Nextera XT DNA Library Preparation Kit (Illumina) and KAPA HiFi HotStart PCR Kit as follows: 1X KAPA HiFi Fidelity Buffer, 0.5 mM MgCl_2_, 0.3 mM KAPA dNTP Mix, 0.3 μM primers, 0.5 U KAPA HiFi HotStart DNA Polymerase, and 5 μL of Nextera XT Index 1 Primers and Nextera XT Index 2 Primers per sample. The procedure was performed as described in the *16S Metagenomic Sequencing Library Preparation* guide. Library products were purified as PCR products. The final DNA concentrations of the purified products were measured using a Qubit 3.0 fluorimeter (Thermo Fisher Scientific) and validated with the Agilent 2100 Bioanalyzer (Agilent Technologies, Santa Clara, CA, USA). The purified products were diluted to a final concentration of 4 nM and pooled. The pool was denatured and sample loaded, as per the *16S Metagenomic Sequencing Library Preparation* guide, at a final concentration of 7 p.m. The 16S rRNA gene libraries were sequenced with 2 × 300 paired-end reads using the Illumina MySeq system (Illumina).

#### Statistics, Sequence Processing and Bioinformatics Data Analysis

The non-parametric Mann–Whitney test was used to make comparisons between groups using Graphpad Prism (GraphPad software v6.0, La Jolla, California, USA). Survival curves were created using the product limit method of Kaplan and Meier and were compared using the log-rank test. Body weight increase slopes were compared using linear regression test. Statistically significant differences are designated as follows: ^*^p < 0.05; ^**^p < 0.01; ^***^p < 0.001; ^****^p < 0.0001.

#### Immune Response Analysis

A three-way ANOVA was used to assess the effect of diet, infection and BCG vaccination on the immune response in lungs. A logarithmic transformation was applied to normalize the cytokine concentrations. In order to obtain equally sized samples, the missing value in the ND-MCI group was replaced by the mean for the group. Holm–Sidak multiple comparisons were performed using the means of groups differing in only one factor. Plotting and statistical analysis were carried out using Graphpad Prism (GraphPad software v7.0, La Jolla, California, USA).

The immune response in lungs was also analyzed by Principal Component Analysis (PCA) using RStudio (version 1.1.463). PC1 and PC2 represent the two principal components that contribute to most of the variance. The direction of the arrows and their lengths represent the contribution and strength of each principal component. Cytokine concentration levels were scaled to unit variance.

#### Bacterial Diversity Analysis

The resulting read files were analyzed using mothur pipeline (version 1.39.5). Paired-end reads were joined and quality controlled. Sequences were aligned using the SILVA reference database. Those alignments that did not fit exactly with our region of interest were removed. Sequences were pre-clustered and chimeric sequences were identified and removed, then taxonomically classified using RDP (ribosomal database project). Sequences classified as chloroplasts, mitochondria, archaea, and eukaryote were eliminated. The distances between sequences were calculated with a distance cut-off of 0.03. After creating the distance matrix, sequences with >97% similarity were clustered into operational taxonomic units (OTUs). These OTUs were filtered and only those that had more than 0.1% relative abundance and were in at least two samples were conserved. Each OTU was assigned to a taxonomic classification. OTUs were classified into the last taxonomic rank possible, with genus being the furthest rank allowed by the method used. Rarefaction curves, diversity and richness indexes and distances between samples were calculated and plotted using RStudio (version 1.0.143). Diversity was quantified using the Bray–Curtis dissimilarity test and plotted using *non-metric multidimensional scaling* (NMDS). Beta diversity (Shannon's, Simpson's, and Inverse Simpson's index) was calculated to analyze differences in diversity between samples.

## Results

### HFD Determines the Spectrum of Body Weight Before and After Infection

**Figure 1:**
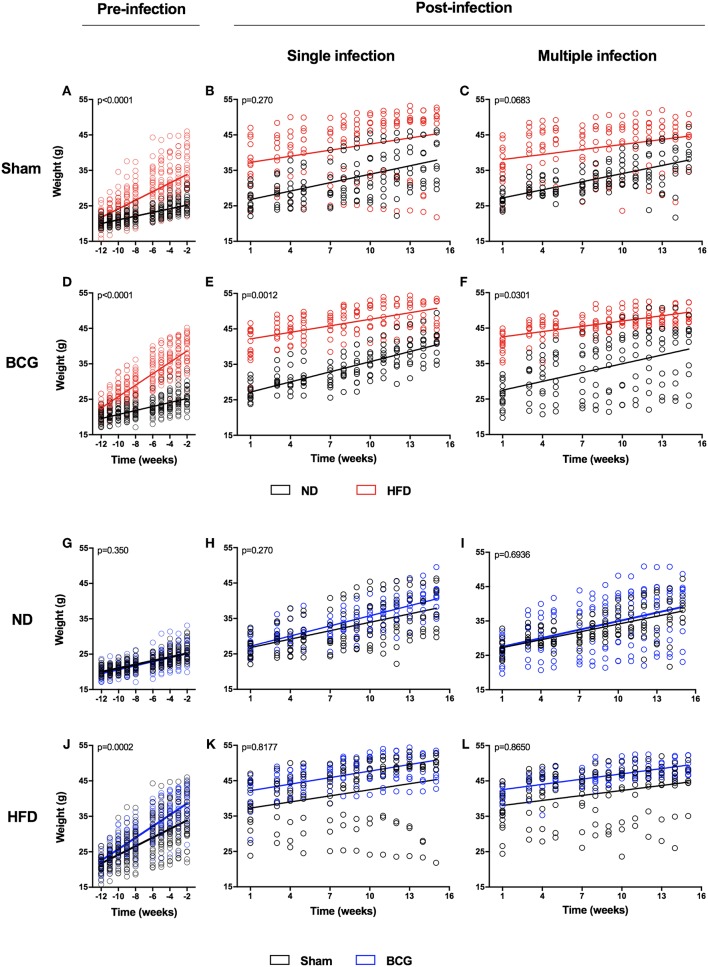
Weight evolution during the experiment in pre- and post-infection status. Weight evolution of the same experiment is represented by comparing ND and HFD **(A–F)** and Sham and BCG **(G–L)**. Panels represent: pre-infection and sham **(A)**, SI and sham **(B)**, MCI and sham **(C)**, pre-infection and BCG **(D)**, SI and BCG **(E)**, MCI and BCG **(F)**, pre-infection and ND **(G)**, SI and ND **(H)**, MCI and ND **(I)**, pre-infection and HFD **(J)**, SI and HFD **(K)**, MCI and HFD **(L)**. Linear regression are represented together with *p*-values. Slopes' values are included in Table S1.

The animals' weight was recorded weekly throughout the study. HFD was found to significantly increase body weight at 2 weeks after starting the diet. Before *Mtb* challenge there was a clear difference in the slope of the body weight increase between ND and HFD (Figures 1A–F; Table S1; Figure S1). However, this difference in the growth slope disappeared suddenly after the challenge for both SI (Figures 1B,C) and MCI (Figures 1E,F), with HFD exhibiting a sudden deceleration. BCG vaccination significantly increased body weight in the HFD group just 6 weeks after starting the diet (Figures 1G–L; Figure S1). Animals also showed glucose intolerance and increased cholesterol and HDL (Figure S2).

### BCG Generates Lower Protection in Mice Fed With HFD in Terms of BL in Lung Tissue

The BL in lungs was evaluated at weeks 3, 4, and 16 p.i. No statistically significant differences were found in terms of BL when comparing diets, although HFD-BCG animals tended to have higher medians (Figure S3). The type of challenge is a determinant factor in BL, and was found to be higher in almost all MCI groups (Figure S4).

**Figure 2:**
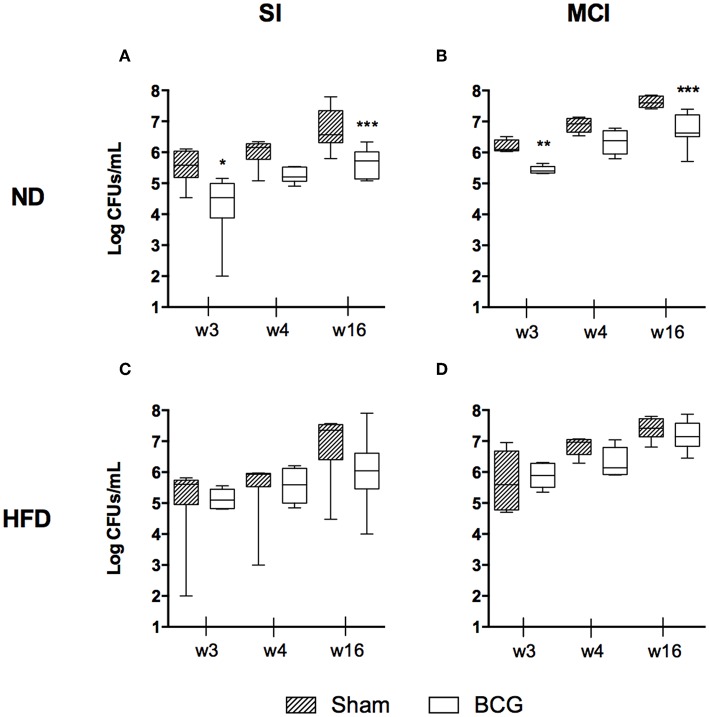
Bacillary load progression at different end time points (w3, w4, and w16) are shown as log CFUs/ml. Each panel compares sham and BCG vaccinated groups: ND and SI **(A)**, ND and MCI **(B)**, HFD and SI **(C)**, HFD and MCI **(D)**. Box and whiskers plots show the minimum, first quartile, median, third quartile and maximum values. *p < 0.05, **p < 0.01, ***p < 0.001; Mann–Whitney test.

As expected, BCG decreased BL by ~1 log in the groups fed with ND. This effect was maintained over time (Figures 2A,B). This improvement was not observed in HFD groups for either the SI or MCI challenge (Figures 2C,D). It should be noted, however, that BCG vaccination was administered at the same time as HFD, before having differences in weight between groups.

### Early Infiltration Induced by BCG Does Not Preclude a Long-Term Reduction in Pathology Under HFD

**Figure 3:**
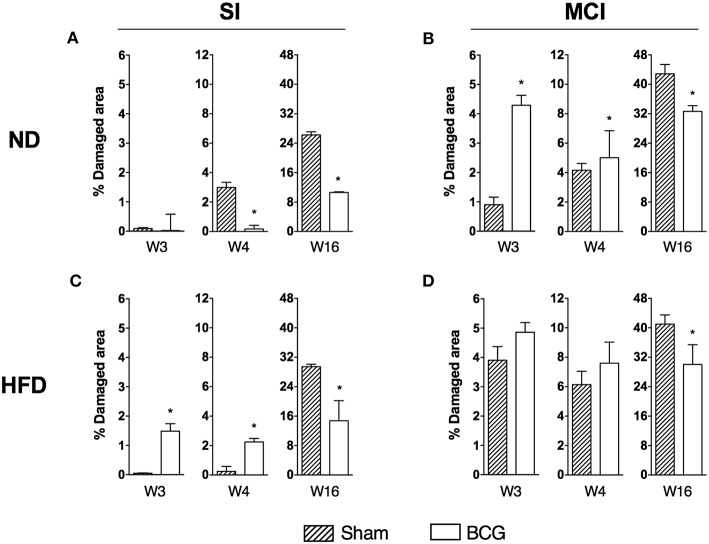
Progression of lung disease at different end time points (w3, w4, and w16) is shown as the percentage of damaged area. Each panel compares sham and BCG vaccinated groups: ND and SI **(A)**, ND and MCI **(B)**, HFD and SI **(C)**, HFD and MCI **(D)**. Bar plots show median with range. *p < 0.05; Mann–Whitney test.

Analysis of the histometry revealed different trends in the acute (weeks 3 and 4) and chronic (week 16) phases of infection (Figure 3).

In the early stages of infection, and after SI, BCG-vaccinated animals exhibited less infiltration than sham-vaccinated animals if fed with ND. However, the opposite was observed in HFD-fed animals. In contrast, under MCI and ND conditions, vaccination resulted in larger damaged areas when compared to control animals. Under MCI and HFD conditions, although vaccinated mice exhibited more infiltration, this difference was not statistically significant as the sham group also presented a large damaged area. In the chronic phase, BCG induced less infiltration than sham-vaccinated mice under all conditions.

**Figure 4:**
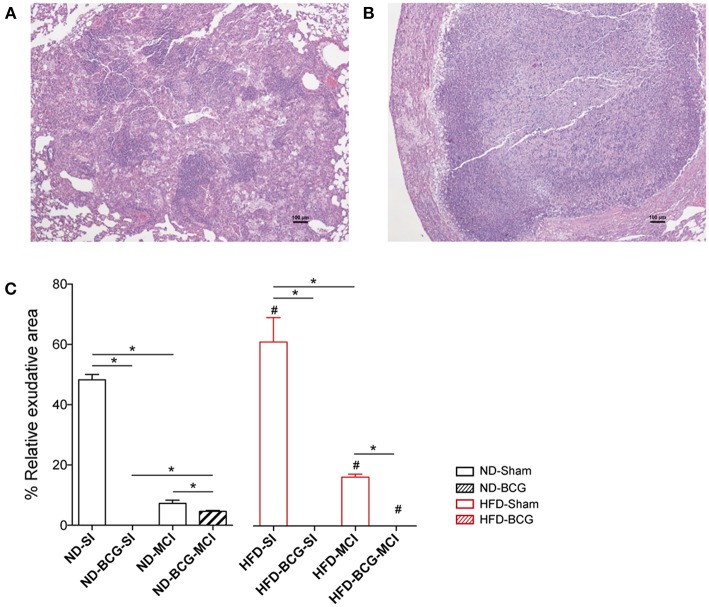
Quality of the lesions found at week 16 post-infection. The tissue sections show a proliferative **(A)** and an exudative **(B)** lesion stained with H/E. **(C)** Relative percentage of exudative lesions at week 16. Black bars represent ND groups while red bars represent HFD groups. Hatched bars in each type of diet represent vaccination Bar plots show median with range. Asterisk indicates differences between vaccination and infection in each diet; hashtag indicates differences between diets. *p < 0.05, ^#^*p* < 0.05; Mann–Whitney test.

Lesions were classified as proliferative or exudative depending on their quality. Exudative lesions were present in all sham groups and were predominant after SI. Most of the lesions in BCG-vaccinated animals were proliferative, with exudative lesions being present only in ND-MCI mice. Amongst the sham groups, HFD exhibited a higher percentage of exudative lesions than ND (Figure 4). Given the absence of exudative lesions in the acute phase, this measurement was only performed at week 16.

### Cytokine Profile Analysis

Pro-inflammatory cytokines (IFN-γ, IL-2, IL-17, IL-12, IL-6, and TNF-α), neutrophil recruitment chemokines (CXCL1 and CXCL5) and anti-inflammatory cytokines (IL-10 and TGF-β) were analyzed at week 4 p.i. in lung homogenates.

Table 3 shows the *p*-value summary of the three-way ANOVA results. Infection was the factor responsible for the largest variation between cytokines and chemokines (significant in all except IL-2 and TGF-β). Similarly, IL-2 and IL-12 levels were significantly affected by BCG vaccination. Thus, the two dietary conditions alone had no significant effect on the cytokine profile. Some of the cytokines analyzed presented an interaction between different factors. For more detailed information, each individual value is illustrated in Figure S5. Briefly, pro-inflammatory cytokines were present in higher concentrations in MCI animals. In the case of IFN-γ, TNF-α, and IL-17, this increase was higher in mice fed with HFD. In fact, among SI, ND mice had higher levels of these immune mediators, with the opposite being found for MCI. The same pattern was observed for CXCL1 and CXCL5. The levels of anti-inflammatory IL-10 or TGF-β did not differ significantly between the experimental groups.

**Table 3:** *P*-value summary of three-way ANOVA from cytokines quantified in lungs (*p < 0.05; **p < 0.01; ***p < 0.001; ****p < 0.0001).

**Source of variation**	**IFN-γ**	**IL-2**	**IL-6**	**IL-10**	**IL-12**	**CXCL5**	**IL-17**	**CXCL1**	**TNF-α**	**TGF-β**
Infection	****	ns	****	**	****	*	****	****	****	ns
Diet	ns	ns	ns	ns	ns	ns	ns	ns	ns	ns
BCG	ns	*	ns	ns	*	ns	ns	ns	ns	ns
Infection × Diet	***	ns	ns	ns	ns	**	*	*	**	ns
Infection × BCG	**	ns	ns	ns	ns	ns	**	*	ns	ns
Diet × BCG	*	****	ns	ns	ns	ns	ns	*	**	ns
Infection × Diet × BCG	ns	**	ns	ns	ns	ns	ns	ns	ns	ns

**Figure 5:**
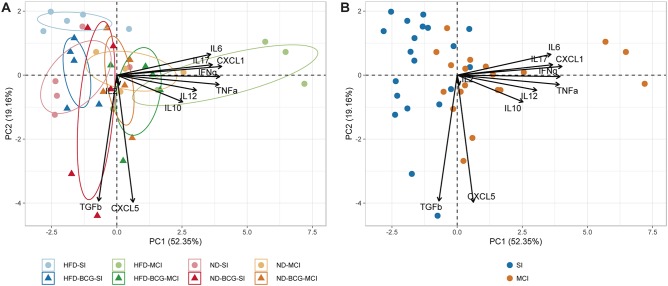
Principal component analysis of inflammatory mediators. **(A)** Representation of animals according to experimental groups with 95% confidence ellipses. **(B)** Samples colored by type of infection.

A PCA was performed for all inflammatory mediators (Figure 5). The first two components explain 71.51% of the variation among samples, with the main contributions to PC1 coming from CXCL1, IFN-γ, TNF-α, IL-17, and IL-6. PC2, in contrast, is mostly explained by CXCL5 and TGF-β. The unvaccinated HFD-MCI animals presented higher PC1 values, whereas HFD-SI animals had higher levels of PC2. As concluded from the three-way ANOVA analysis, the type of infection was the factor with the greatest influence on the inter-sample variation.

### BCG Vaccination Does Not Increase Survival Under HFD, Whereas NI Does

**Figure 6:**
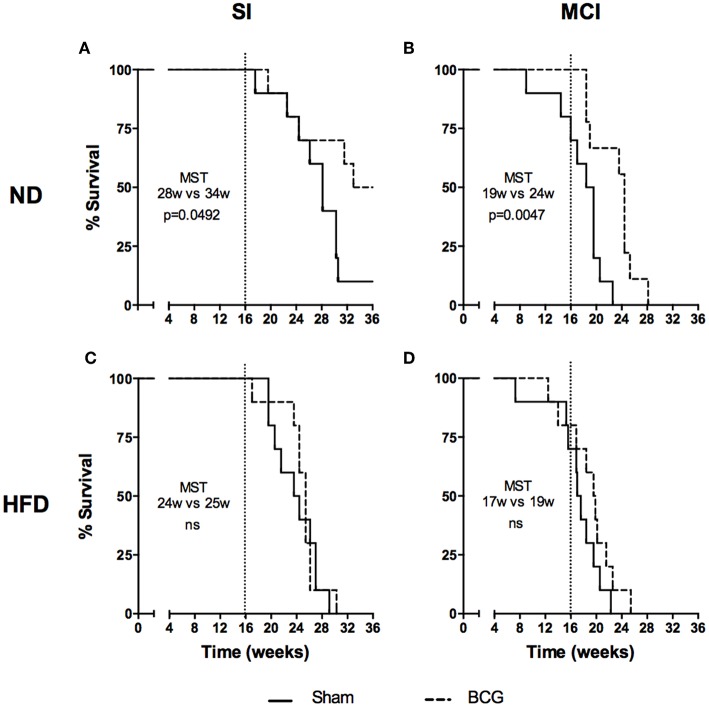
Evolution of survival after *Mtb* infection for the first experiment. Each panel compares sham and BCG vaccinated groups: ND and SI **(A)**, ND and MCI **(B)**, HFD and SI **(C)**, HFD and MCI **(D)**. Median survival times and *p*-values are indicated in each panel. Log-rank test.

**Figure 7:**
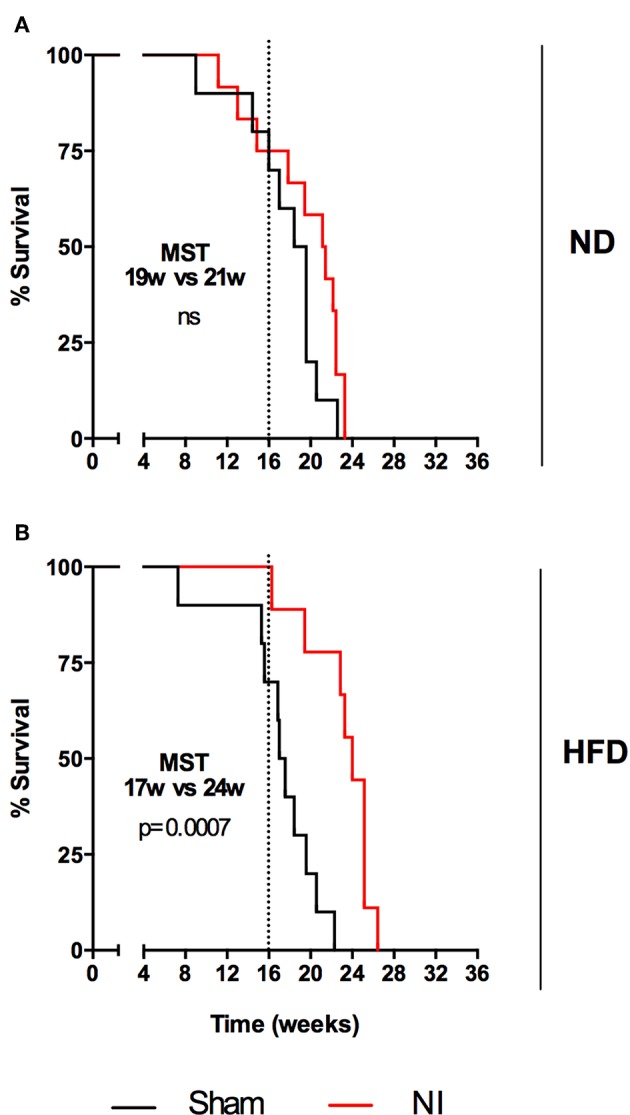
Evolution of survival after *Mtb* infection for the second experiment. Each panel compares sham and NI groups: ND **(A)** and HFD **(B)**. Median survival times and *p*-values are indicated in each panel. Log-rank test.

Differences in survival rates were analyzed using the Log-rank test (Figure 6). Irrespective of their vaccination status, MCI mice had lower survival rates than their SI counterparts, and HFD always presented a lower median survival time (MST). BCG was able to significantly increase survival rates irrespective of the type of challenge under ND (MST from 28 to 34 weeks for SI and MST from 19 to 24 weeks for MCI) but not under HFD feeding. Interestingly, NI induced in animals following antibiotic treatment after SI, until no detectable CFU was achieved, was protective in HFD but not in ND (Figure 7).

### HFD-Induced Obesity and Chemotherapy Reduce Gut Microbiota Diversity

**Figure 8:**
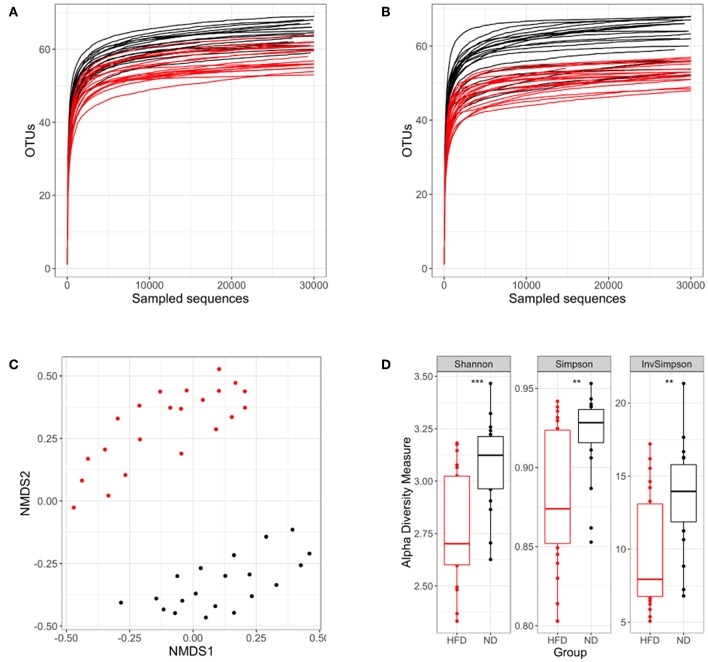
Analysis of the microbiota diversity based on 16S rRNA sequencing. In each panel the ND group is colored in black and the HFD group in red. Rarefaction curves at week 16 **(A)** and week 28 **(B)** after starting the different diets are represented. **(C)** NMDS ordination in samples following different diets. **(D)** Shannon, Simpson and inverse Simpson diversity indexes. **p < 0.01, ***p < 0.001; Mann–Whitney test.

The gut microbiota studies were performed at weeks 4 and 16 post-infection, corresponding to weeks 16 and 28 from the beginning of the diet. In our study, OTUs can be considered as the species found in fecal samples. Given the same number of reads sequenced, a smaller number of OTUs were found in HFD samples at both time points (Figures 8A,B), thus suggesting a lower microbial diversity.

We then assessed the dissimilarity between both diets in terms of microbiota composition using an NMDS plot and the Bray–Curtis dissimilarity index. Samples were grouped according to the type of diet, showing that microbiota composition differs substantially between diet conditions (Figure 8C). Other comparisons were performed to evaluate the influence of BCG vaccination and infection dose on microbiota composition. This analysis was performed separately for the ND and HFD groups, showing no significant differences (Figure S6).

Once the dissimilarity between diets had been confirmed, Shannon, Simpson, and Inverse Simpson indexes were used to demonstrate the existence of real differences in microbiota diversity between the different nutritional statuses. These indexes showed that ND mice had a statistically significantly higher diversity than HFD groups (Figure 8D).

**Figure 9:**
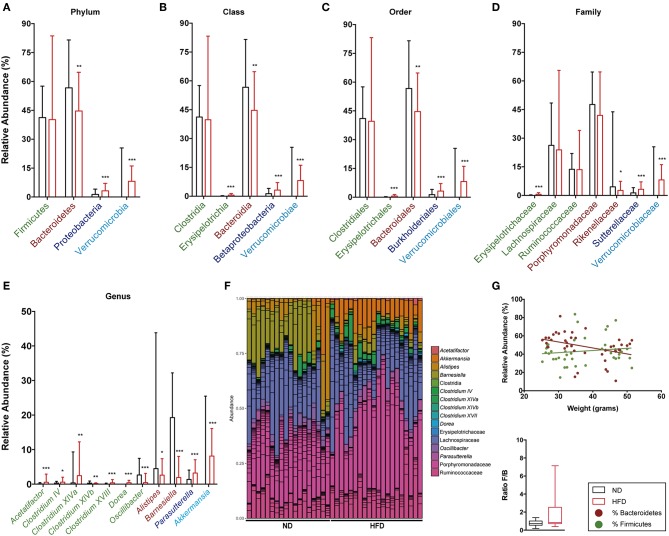
Taxonomic composition of the intestinal microbiota based on 16S rRNA sequencing at week 16 after starting both diets. Analysis of the relative abundance of the most abundant phylum **(A)**, class **(B)**, order **(C)**, family **(D)**, and genus **(E)** in ND vs. HFD. **(F)** Bar plot representation of the OTUs of each sample according the taxonomic classification. **(G)** Regression plot of Firmicutes and Bacteroidetes phyla against animal weight and analysis of the F/B ratio in ND and HFD. *p < 0.05, **p < 0.01, ***p < 0.001; Mann–Whitney test.

**Figure 10:**
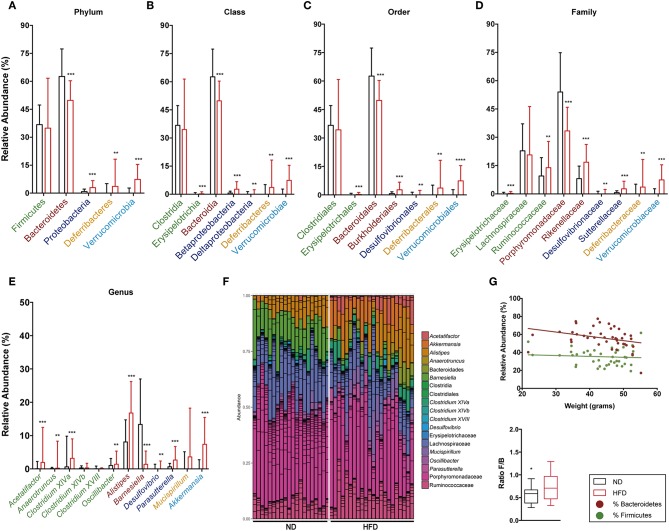
Taxonomic composition of the intestinal microbiota based on 16S rRNA sequencing at week 28 after starting both diets. Analysis of the relative abundance of the most abundant phylum **(A)**, class **(B)**, order **(C)**, family **(D)**, and genus **(E)** in ND vs. HFD. **(F)** Bar plot representation of the OTUs of each sample according the taxonomic classification. **(G)** Regression plot of Firmicutes and Bacteroidetes phyla against animal weight and analysis of the F/B ratio in ND and HFD. *p < 0.05, **p < 0.01, ***p < 0.001, ****p < 0.0001; Mann–Whitney test.

The relative abundance of Firmicutes and Bacteroidetes phyla and the ratio between them were analyzed at both time points, namely week 16 (Figures 9A,G) and week 28 (Figures 10A,G). This analysis showed an inverse correlation between the relative abundance of Bacteroidetes and the animals' weight, with the F/B ratio being higher in HFD mice due to the reduction in Bacteroidetes.

The detailed gut microbiota composition results are shown in Figure 9 (week 16) and Figure 10 (week 28). Analysis of the most abundant families showed a significant increase in Ruminococcaceae and Rikenellaceae, and a reduction in Porphyromonadaceae with HFD at week 28. Among the genera from the Firmicutes phylum, mice fed with HFD showed a higher abundance of *Acetatifactor, Anaerotruncus, Clostridium* XIVa, and *Oscillibacter* at week 28. In contrast, analysis of genera from the Bacteroidetes phylum showed that HFD mice had a lower abundance of *Alistipes* at week 16 than ND, whereas the opposite was the case at week 28. The levels of the *Barnesiella* genus are markedly higher in ND-fed animals at all time points, whereas *Parasuterella* (Proteobacteria) is higher in HFD. It is interesting to note the predominance of the *Akkermansi*a genus at both time points, and the appearance of *Mucispirillum* at the last time point, in HFD-fed mice.

**Figure 11:**
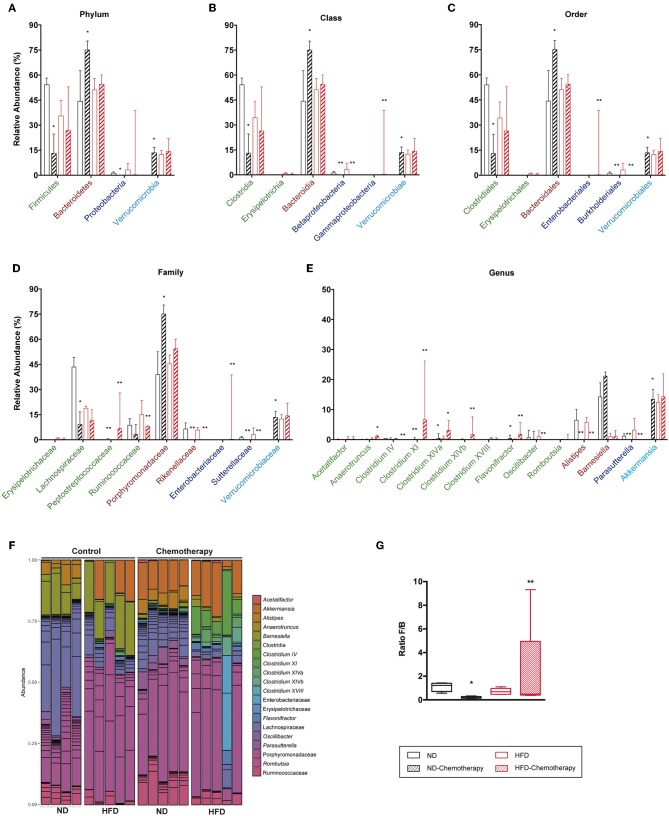
Taxonomic composition of the intestinal microbiota based on 16S rRNA sequencing between different diets and under chemotherapy. Analysis of the relative abundance of the most abundant phylum **(A)**, class **(B)**, order **(C)**, family **(D)**, and genus **(E)**. **(F)** Bar plot representation of the OTUs of each sample according the taxonomic classification. **(G)** Analysis of the F/B ratio. *p < 0.05, **p < 0.01; Mann–Whitney test.

The effect of chemotherapy used to induce NI was also assessed on gut microbiota (Figure 11). Results indicated that chemotherapy decreased the F/B ratio in ND due to both a decreased abundance of Firmicutes and increased levels of Bacteroidetes. Interestingly, chemotherapy showed the same trend for HFD, although the differences were not statistically significant. In the Firmicutes phylum, Lachnospiraceae and Ruminococcaceae were reduced with chemotherapy in both diets, whereas for Bacteroidetes, the Porphyromonadaceae family increased with chemotherapy. Finally, the genus *Akkermansia* was substantially increased with chemotherapy in ND.

## Discussion

To the best of our knowledge, this is the first time that diet-induced obesity has been studied in an experimental TB model. We have chosen to test the impact of an HFD as obesity inducer as this is the most robust and best characterized model for inducing metabolic syndrome and glucose intolerance, which are the main factors leading to DM2 (14). Although our model was able to induce a marked increase in body weight, we did not observe a constant hyperglycemia and animals had no need for insulin treatment. Indeed, this is the current situation for millions of citizens with this syndrome who will go on to develop DM2 (25).

These variables are introduced in the context of the C3HeB/FeJ mouse, a unique strain that is able to reproducethe spectrum of human lesions and is considered to best reflect the progression from infection to disease (26). This progression differs from the general tolerance toward *Mtb* infection shown by inbred mice, which only develop proliferative lesions (27, 28).

In view of recent data, our model is more complex than we initially thought. Dalby et al. (29) have demonstrated that refined diets reduce gut microbiota diversity and increase the F/B ratio irrespective of obesity or dietary fat. They showed that a refined low fat diet is also able to induce dysbiosis, with no link to obesity or hyperglycemia. In any case, a high F/B ratio favors glucose intolerance and is thus linked to an impaired immune response (14). As such, we have two independent factors that can explain the increased progression toward TB. First, although metabolic syndrome and DM2-related obesity, which are clearly linked to an immune dysfunction, have been observed in other models, this is not the case in ours. Pre-feeding with HFD for 8 weeks in CD-1 mice reduced the lethality of *Trypanosoma cruzi* infection by 35% (7). Secondly, dysbiosis and an increase in the F/B ratio. This has also been linked to a decreased resistance to disease in a study comparing wild mice with lab mice (30). On the other hand, *Mtb* infection has consistently been shown to be linked to a reduction in gut microbiota diversity (15, 17). Our data show that HFD aggravates this tendency, thus resulting in a lower survival in animals fed with HFD.

Although there were no differences in BL between the diets, BCG only had a positive effect in ND mice, thus revealing a diminished protective capacity under HFD conditions. To the best of our knowledge, and with the exception of some experiments with a Beijing strain challenge (31), this is the first time that no protective effect has been seen upon BCG vaccination in mice in terms of BL reduction. However, these strains are related to the induction of a different pattern of immune response after triggering an IFN type I response (32), therefore further studies are required to determine whether HFD also induces such a response.

Histometry studies shortly after the SI challenge showed an unusual reaction for the HFD group as these mice exhibit a stronger reaction after vaccination with BCG than with sham, in contrast to what occurs in ND animals. Even when this is not maintained throughout the chronic phase, it seems to be indicative of a delayed immune response. Moreover, MCI mice (both BCG and sham vaccinated) exhibit a stronger infiltration at all the time points studied, although a higher percentage of damaged area at week 3 after BCG vaccination in ND reflects a better immune response due to the vaccination. This different reaction in HFD correlates with the immune impairment of the response against TB linked to obesity and DM2, as seen above, although this chronic increase in the pro-inflammatory response does not result in the expected clear histological phenotype in terms of increasing the number of exudative lesions. This is in accordance with the intensity of the risk factor shown by DM2 (around ×3), which is much lower than the risk factor for immunosuppression by anti-TNF or AIDS (around ×100), where a clear phenotype can be seen.

As described by several authors, aerosol infection is able to induce two kinds of lesions in this model, namely proliferative lesions, which are characterized by a slow progression and spreading via infected foamy macrophages, and exudative lesions, in which neutrophils accumulate at the periphery, thereby fueling the extracellular bacillary growth that finally results in large-scale central necrosis (20, 26, 33, 34). Surprisingly, our analysis of the quality of the lesions revealed larger exudative regions in SI animals than those MCI infected. As we are currently unable to explain this phenomenon, further studies in this regard are required.

The pulmonary cytokine profile showed that, for SI animals, there is a more pro-inflammatory profile in ND than in HFD fed mice, irrespective of vaccination status, thus confirming the delay in the onset of the immune response in HFD seen in the histometry. However, this pattern reverts when animals are challenged with MCI, thus receiving far higher bacillary counts. This strong pro-inflammatory response in HFD mice has no advantage in terms of BL reduction, a fact than may reflect a higher basal pro-inflammatory milieu. This could also explain the pathology results.

Data published by Rosshart et al. (30) regarding the ability of natural microbiota, in terms of diversity and F/B ratio, to benefit a balanced systemic and local inflammatory response, which in this case would have more similarities to ND than HFD fed mice, merit more in-depth studies, including mice fed with refined low fat diets (29). This is an important issue given the recent paper by Maji et al. (19), which found a higher diversity of the intestinal microbiome in TB patients compared with controls, thus resulting in a higher presence of short-chain fatty acids. These authors claim that these products, especially butyrate, have a strong anti-inflammatory effect after stimulating Tregs in the gut, thus promoting IL-10 production and facilitating a chronic infection, which contradicts the concept of Rosshart et al. (30). Indeed, as highlighted by the authors, there is an important confusing factor in that study, namely the BMI, which is significantly lower in TB patients. It is well-known that a low BMI has a major impact in TB and other diseases by inducing a global immune dysfunction as a consequence of protein-energy undernutrition, which impairs T-lymphocyte-mediated responses (35).

In our model, HFD caused gut microbiota dysbiosis, thus inducing a pro-inflammatory milieu that limits the bacteriome diversity. This caused an increase in the F/B ratio. These are the most important phyla in humans, with Bacteroidetes being reduced due to obesity and subsequently being restored, along with a reduction in Firmicutes, after losing body weight on a diet (36). The question now arises as to how this dysbiosis influences the fate of TB progression. The recent study by Rosshart et al. (30) links a high diversity and low F/B ratio to the promotion of host fitness and limited inflammatory responses against influenza A and colorectal tumorigenesis. This is coherent with our findings, which also point to a sort of “neutral effect” caused by the abundance of Porphyromonadaceae family, and specifically *Barnesiella*, found under ND conditions.

In our system, analysis of the microbiota showed differences with previous data obtained in mice fed with HFD. These differences are probably related to both *Mtb* infection and the time of feeding, as in our case the analysis was done after 16 and 28 weeks of feeding, which is far longer than the usual 8–12 weeks reported previously (14, 29).

With regard to the Firmicutes phyla, ND presented lower levels (at week 28) of Ruminococcaceae, which is related to the production of short-chain fatty acids (37), thus triggering an anti-inflammatory response. It is interesting to note that the epitope expressed on the flagellin of the Lachnospiraceae, which becomes the most important Firmicutes family in both diets, is a potent inducer of Tregs and TGF-β in dendritic cells in mice (38, 39). Lachnospiraceae increased significantly in mice under stress. In this study, stress caused an increase in systemic corticoid, IFN-γ and IL-17 levels, as well as mucosal injury (40). This is coherent with the stress induced by TB infection regardless of diet. At a genus level, and although they only represented a small percentage of the total microbiota, HFD presented an increase in *Clostridium XIVa*, which promotes the expansion of Tregs (39), as well as *Anaerotruncus* and *Acetatifactor*, which have been related to arthritis progression in mice, thus suggesting a pro-inflammatory response (41). Interestingly, we have also detected a total absence of the Lactobacillaceae family in C3HeB/FeJ mice. This is a relevant factor as, according to Winglee et al. (15), this is the predominant family (around 50% of relative abundance) in *Mtb*-infected C57BL/6 mice. Curiously, recent data from Namasyvayam et al. (16) in *Mtb*-infected C57BL/6J-CD45a(Ly5a) mice also found this family, but in a very low relative abundance (around 3%). As such, this may be a critical point in the gut microbiome of C3HeB/FeJ as it may be determinant for triggering the strong inflammatory response that causes exudative lesions. Lactobacillaceae has also been related to a decrease in the inflammatory response, and thus immune balance, although this proposal remains controversial as it was reported in the context of inflammatory bowel disease (37).

The most remarkable differences caused by diet were seen in the Bacteroidetes phylum. Thus, HFD-fed mice showed significantly lower levels of the *Barnesiella* genus at both time points, along with an increase in the *Alistipes* genus with time. These changes may be crucial in the different evolution experimented by both groups. Thus, the most abundant family in both diets is Porphyromonadaceae, which is decreased under HFD conditions. Porphyromonadaceae is increased in mice fed with a high-protein/low carbohydrate diet (42) and decreased in mice fed with omega-6-polyunsaturated fatty acids (43). This has been related with the dysbiosis shown in patients with ankylosing spondylitis, a heritable immune-mediated arthropathy (44). The *Barnesiella* genus has proved useful for reducing the colonization of *Enterococcus* in a “neutral way” as it is not thought to be associated with immune development or inflammatory diseases in the intestine (45). In contrast, *Alistipes* is the most significant over-represented taxon related to frailty in the elderly (46) and is found to be increased under HFD conditions (47). Overall, it appears that colonization with the Porphyromonadaceae family, and in particular the *Barnesiella* genus, may have a positive impact on reducing the pro-inflammatory milieu in the gut.

With regard to Proteobacteria, HFD caused a sustained increase in the genus *Parasutterella*, which is precisely the opposite to what was found in C57BL/6 (47). Additional microbiome studies in humans and rats have associated an increased abundance of *Parasutterella* with dysbiosis in the gut, with this genus being increased in the submucosa of individuals with Crohn's disease (48) and in rats with hypertriglyceridemia-related acute necrotizing pancreatitis (49).

Finally, HFD mice had a higher relative abundance of *Mucispirillum* and *Akkermansia* genera. Unlike the majority of commensal bacteria, *Akkermansia* and *Mucispirillum* evade T-independent IgA and elicit a T-dependent response (50). In contrast to our observations, the abundance of *Akkermansia muciniphila* is decreased during obesity and diabetes, and administration thereof can counteract the development of HFD-induced obesity and gut barrier dysfunction. Its effect is increased when given inactivated by pasteurization, a mechanism linked to the increase in mucus production (51). *Mucispirillum schaedleri* has been reported to be elevated in abundance during intestinal inflammation in mouse models, and has been described as a mucus-associated bacterium adapted to the oxidative burst that occurs during inflammation (52).

Another unknown was the effect caused by chemotherapy after aerosol infection. A more significant impact was found for the gut microbiota of ND mice, which could give a clue as to why natural infection was less effective in this group. Animals fed with ND presented a drastic reduction in the F/B ratio with chemotherapy. This is mainly explained by a reduction in Lachnospiraceae for Firmicutes and an increase in Porphyromonadaceae for Bacteroidetes, thus increasing the “neutral” effect of the microbiota. Chemotherapy also markedly promoted the presence of the *Akkermansia* genus in ND, thus increasing T-cell stimulation. In view of these results, we can hypothesize that induction of a protective effect by natural infection might require a certain anti-inflammatory effect. This is coherent with recent findings suggesting that excess effector T-cell stimulation might be deleterious to a protective immune response (53, 54). In HFD mice, in contrast, chemotherapy did not impact the abundance of Firmicutes, with the increase in several *Clostridium* appearing to “compensate” the loss of Ruminococcaceae and Lachnospiraceae. It also failed to impact the abundance of Bacteroidetes and triggered the presence of Enterobacteriaceae, which is difficult to analyze since it did not affect the levels of *Akkermansia*.

Overall, our findings show that HFD accelerates the progression toward active TB. Although we have not been able to discern the exact mechanism of action, our results point to a dysregulation in the ability to react to *Mtb* infection, which appears to slow the appearance of immunity in the early stages post-challenge. HFD-fed animals show a reduced ability to detect low dose infection, while reacting excessively to a high dose, thus leading to a reduced survival. This may be related to the HFD causing early DM2, as supported by a dysbiosis in the intestinal microbiota that promotes an intolerance to glucose. Interestingly, even when natural immunity induced by *Mtb* followed by chemotherapy is able to induce protection, BCG vaccination protection is reduced in terms of BL and survival. This finding highlights the need to further explore the mechanisms of this difference in order to design a better vaccine that can protect against this rapidly growing comorbidity, which is starting to hamper current efforts to control TB.

## Data Availability Statement

The datasets generated for this study are available on request to the corresponding author.

## Ethics Statement

The animal study was reviewed and approved by Hospital Universitari Germans Trias i Pujol (registered as B9900005).

## Author Contributions

P-JC led the project. CV, IC, and P-JC conceived and planned the experiments. LA, PC, JD, YR, GG, MT-P, GT, and EG carried out the experiments. LA, GG, PC, IC, CV, and P-JC contributed to interpretation of the results. LA, CV, and P-JC took the lead in writing the manuscript. All authors contributed to drafting the work and gave final approval for the version to be published. All authors agree to be accountable for all aspects of the work in ensuring that questions related to the accuracy or integrity of any part of the work are appropriately investigated and resolved. All authors provided critical feedback and helped shape the research, analysis, and manuscript.

### Conflict of Interest

The authors declare that the research was conducted in the absence of any commercial or financial relationships that could be construed as a potential conflict of interest.

## Abbreviations

TB: Tuberculosis
BMI: Body mass index
DM2: Diabetes mellitus type 2
Tregs: Regulatory T cells
HFD: High fat diet
F/B: Firmicutes to Bacteroidetes
*Mtb*: *Mycobacterium tuberculosis*
BL: Bacillary load
BCG: Bacillus Calmette-Guérin
SI: Single infection
MCI: Multiple consecutive infections
NI: Natural immunity
CFU: Colony-forming unit
ND: Normal diet
p.i.: Post-infection
OTU: Operational taxonomic unit
NMDS: *Non-metric multidimensional scaling*
MST: Median survival time.
