# Supplementary material for: Influence of Gut Microbiota on Progression to Tuberculosis Generated by High Fat Diet-Induced Obesity in C3HeB/FeJ Mice
Source: Front Immunol. 2019 Oct 18;10:2464. doi: 10.3389/fimmu.2019.02464 (PMC6813253; doi:10.3389/fimmu.2019.02464)
Supplement: Supplementary file 1 [file Data_Sheet_1.docx]

Supplementary Material

High Fat Diet-Induced Obesity accelerates the progression to tuberculosis in C3HeB/FeJ Mice. Influence of the gut microbiota

**Lilibeth Arias, Galo Adrián Goig, Paula Cardona, Manuela Torres-Puente, Jorge Díaz, Yaiza Rosales, Eric Garcia, Gustavo Tapia, Iñaki Comas, Cristina Vilaplana, Pere-Joan Cardona*.**

*** Correspondence:** Pere-Joan Cardona: [pj.cardona@gmail.com](mailto:pj.cardona@gmail.com)

1. **Supplementary Figures and Tables**
   1. **Figures**

**
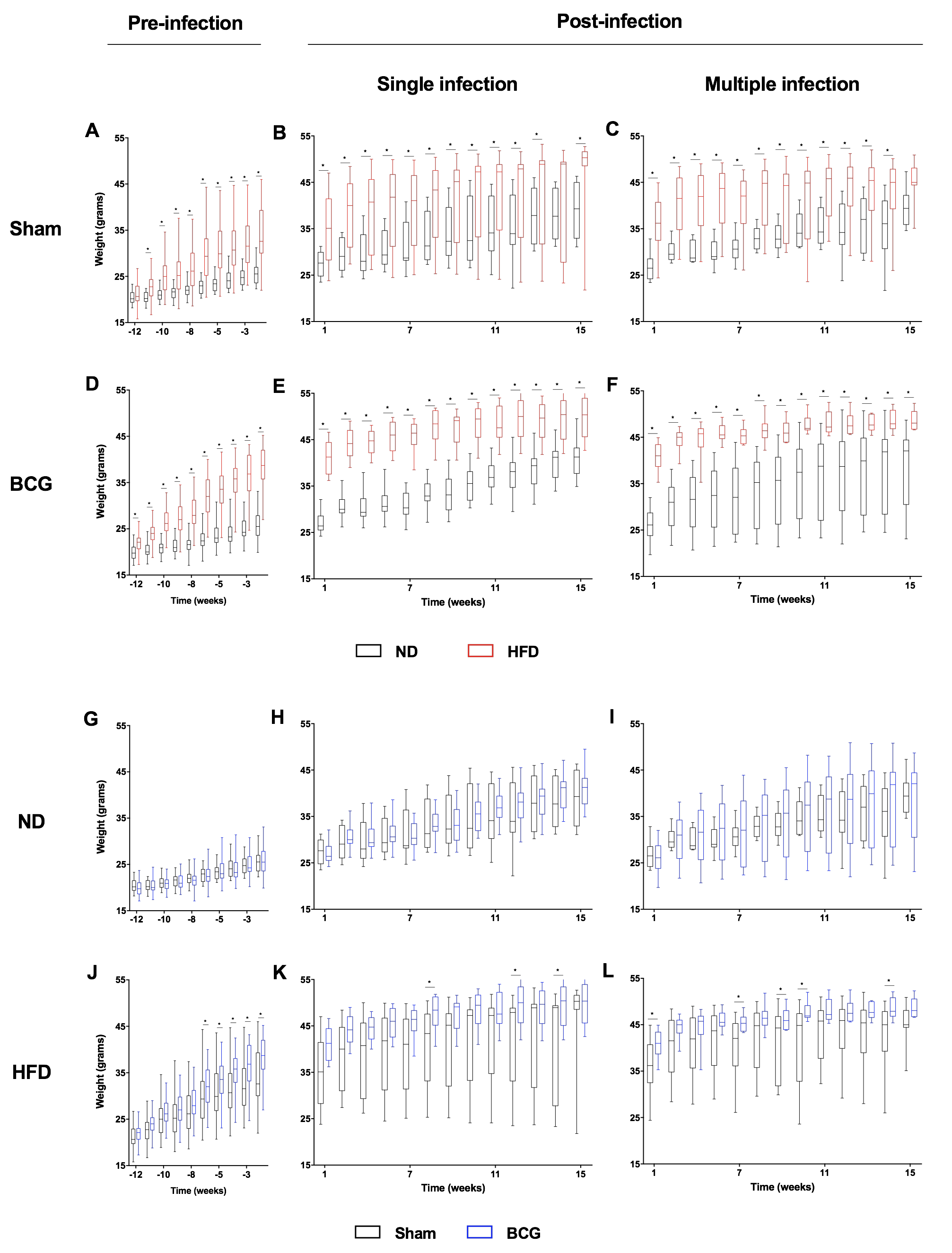
**

**Figure S1.** Box and whiskers plots showing weight evolution during the experiment in pre- and post-infection status. Weight evolution is represented by comparing ND and HFD (**A-F**) and Sham and BCG (**G-L**). Panels represent: pre-infection and sham **(A)**, SI and sham **(B)**, MCI and sham **(C)**, pre-infection and BCG **(D)**, SI and BCG **(E)**, MCI and BCG **(F)**, pre-infection and ND **(G)**, SI and ND **(H)**, MCI and ND **(I)**, pre-infection and HFD **(J)**, SI and HFD **(K)**, MCI and HFD **(L)**. Mann-Whitney test.

**
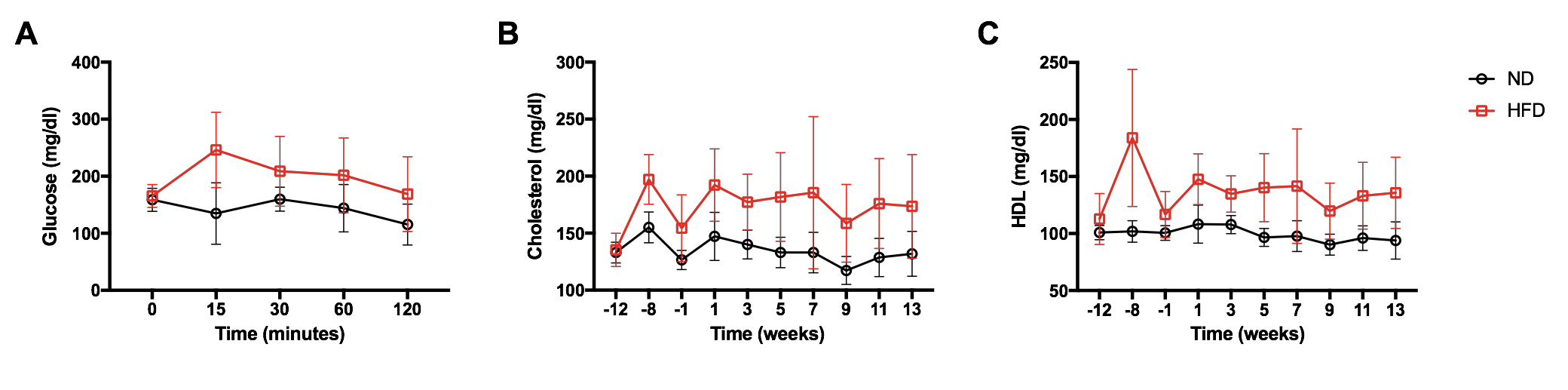
**

**Figure S2.** Glucose tolerance test **(A)**, cholesterol **(B)** and HDL **(C)** in mice given a high fat diet or a normal diet. For glucose tolerance test n=6 animals per group were included. For cholesterol and HDL determination n=18 mice per group were included at each time point. Two way ANOVA test was performed to test diet influence. Diet affects glucose tolerance (*p=*0.0178), cholesterol (*p*<0.0001) and HDL concentrations (*p<*0.0001).

##
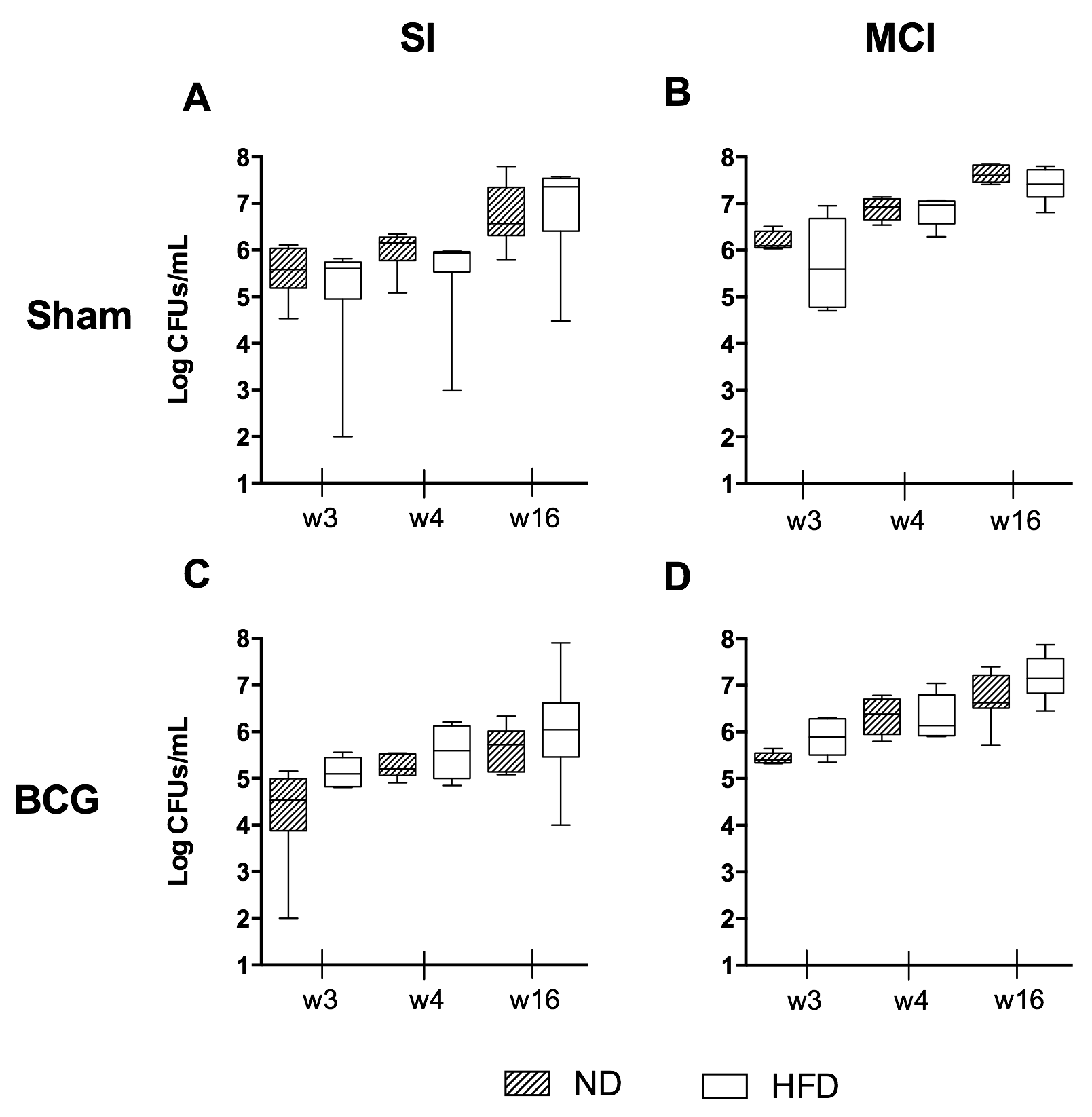


**Figure S3.** Bacillary load progression at different end time points (w3, w4 and w16) are shown as log CFUs/ml. Each panel compares ND and HFD groups: sham and SI **(A)**, sham and MCI **(B)**, BCG and SI **(C)**, BCG and MCI **(D)**. Box and whiskers plots show the minimum, first quartile, median, third quartile and maximum values. Mann–Whitney test.


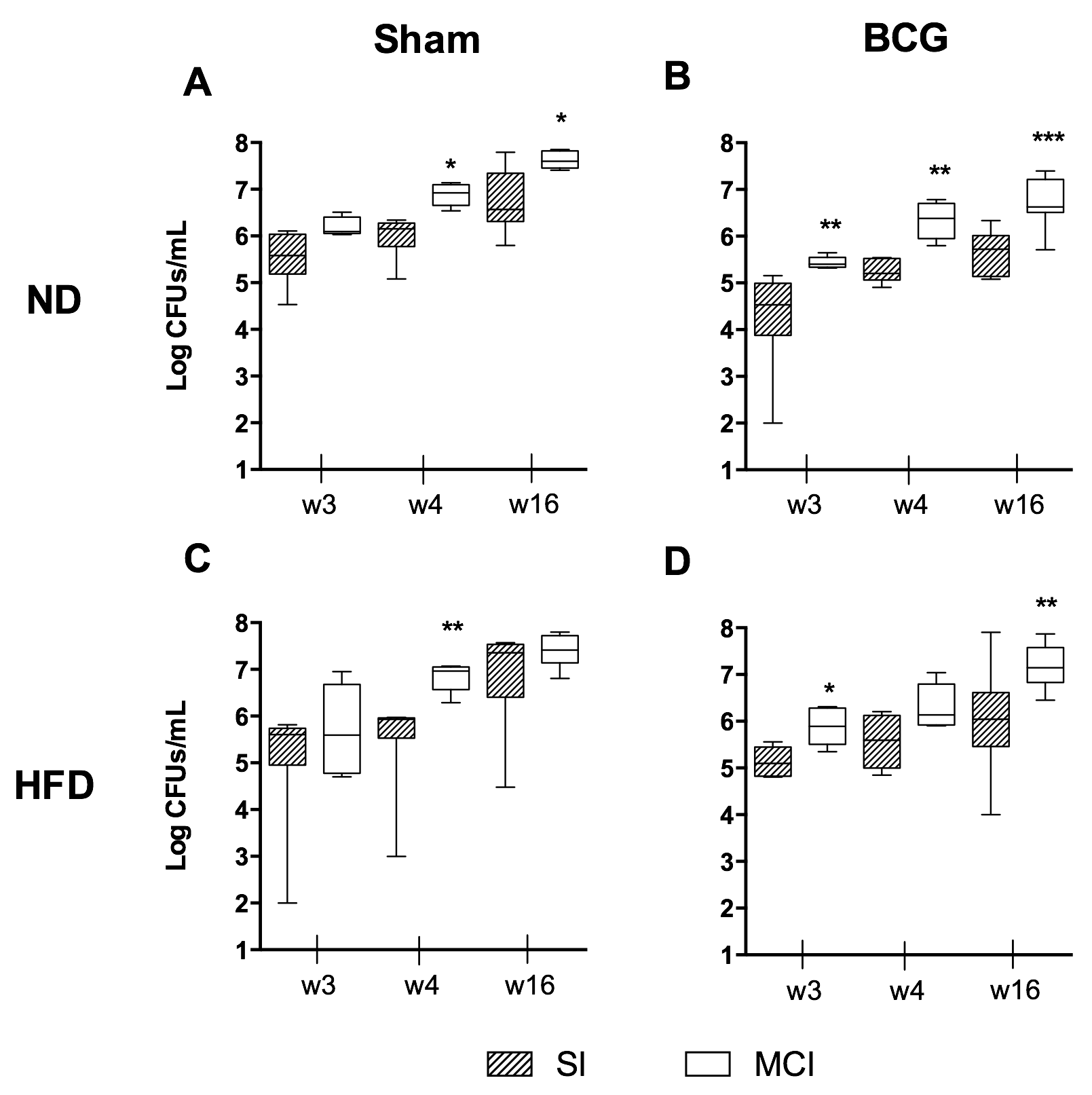


**Figure S4.** Bacillary load progression at different end time points (w3, w4 and w16) are shown as log CFUs/ml. Each panel compares SI and MCI groups: ND and sham **(A)**, ND and BCG **(B)**, HFD and sham **(C)**, HFD and BCG **(D)**. Box and whiskers plots show the minimum, first quartile, median, third quartile and maximum values. Mann–Whitney test.


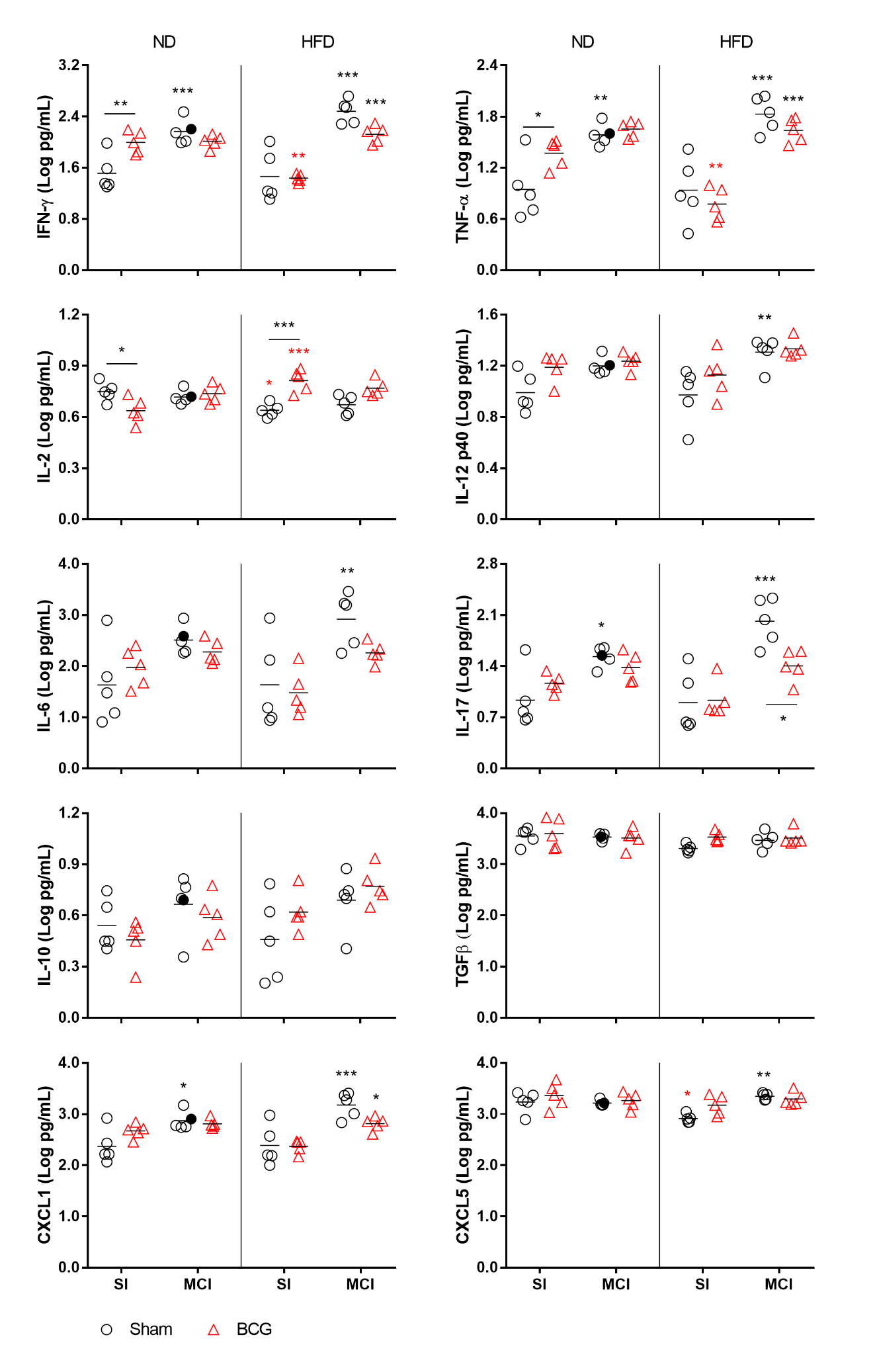


**Figure S5.** Analysis of inflammatory mediators in lung homogenates at w4 post-infection. Results are represented as Log10 of the concentration in pg/ml. Comparisons are differently indicated depending on the experimental condition tested: line and asterisks (vaccination), black asterisks (infection), red asterisks (diet). Holm Sidak’s multiple comparisons test.

**
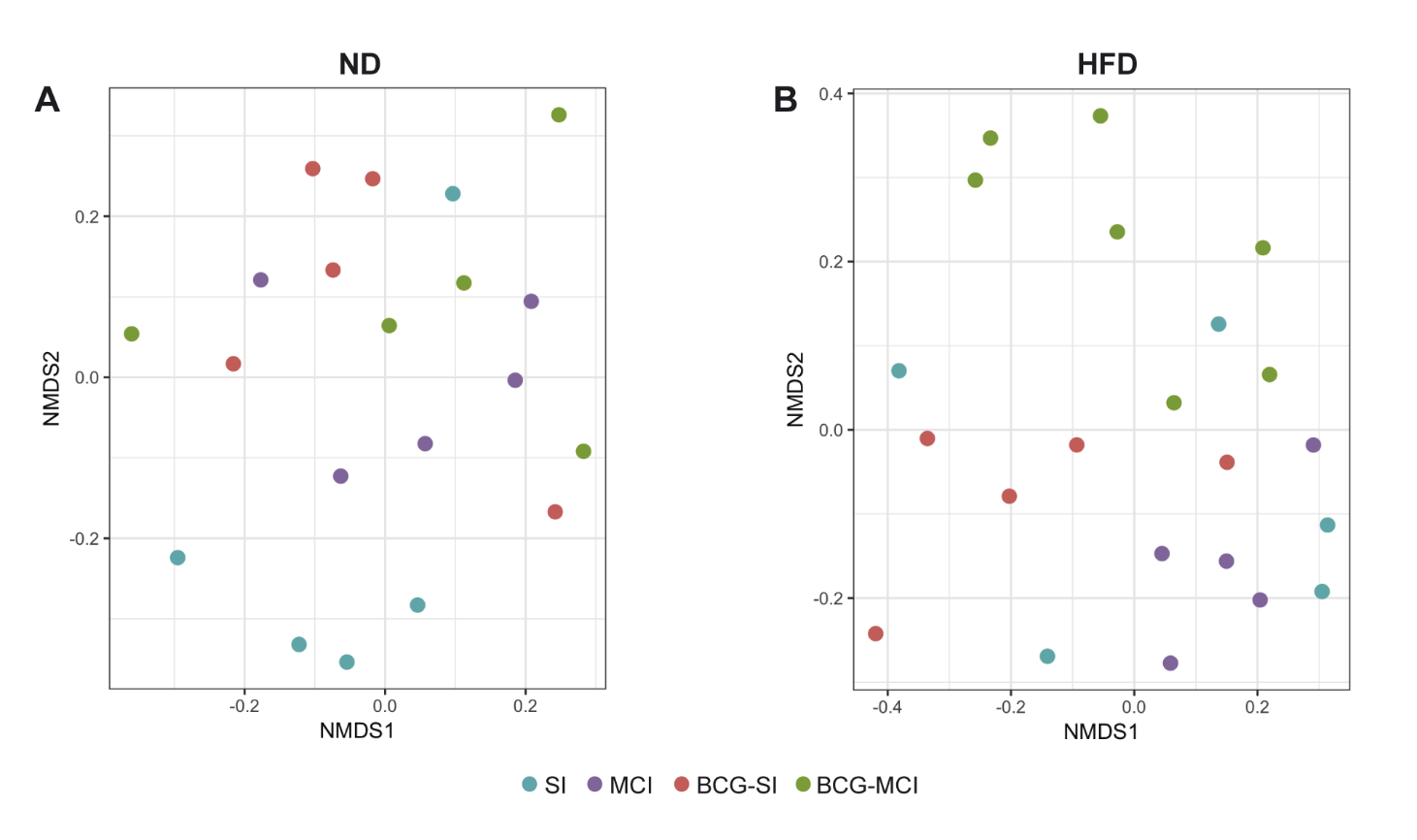
**

**Figure S6.** Analysis of the microbiota diversity based on 16S rRNA sequencing. NMDS ordination was performed separately in each type of diet: ND **(A)** and HFD **(B)**.

- 1. **Tables**

**Table S1.** Comparisons of the pre-infection and post-infection weight increase slopes and their corresponding *p-*values obtained with linear regression test showed in Figure 1

| **Experimental groups** | | | **Slope** | **p-value** |
| --- | --- | --- | --- | --- |
| PRE-INFECTION | Diet | ND | 0.521 | <0.0001 |
|  |  | HFD | 1.213 |  |
|  |  | ND-BCG | 0.565 | <0.0001 |
|  |  | HFD-BCG | 1.61 |  |
|  | Vaccination | ND | 0.521 | 0.350 |
|  |  | ND-BCG | 0.565 |  |
|  |  | HFD | 1.213 | 0.0002 |
|  |  | HFD-BCG | 1.61 |  |
| POST-INFECTION | Diet | ND-SI | 0.793 | 0.270 |
|  |  | HFD-SI | 0.574 |  |
|  |  | ND-MCI | 0.762 | 0.0683 |
|  |  | HFD-MCI | 0.472 |  |
|  |  | ND-BCG-SI | 0.952 | 0.0012 |
|  |  | HFD-BCG-SI | 0.616 |  |
|  |  | ND-BCG-MCI | 0.828 | 0.0301 |
|  |  | HFD-BCG-MCI | 0.496 |  |
|  | Vaccination | ND-SI | 0.793 | 0.270 |
|  |  | ND-BCG-SI | 0.952 |  |
|  |  | ND-MCI | 0.762 | 0.6936 |
|  |  | ND-BCG-MCI | 0.828 |  |
|  |  | HFD-SI | 0.574 | 0.8177 |
|  |  | HFD-BCG-SI | 0.616 |  |
|  |  | HFD-MCI | 0.472 | 0.8650 |
|  |  | HFD-BCG-MCI | 0.496 |  |
